# Collaborative cross mice in a genetic association study reveal new candidate genes for bone microarchitecture

**DOI:** 10.1186/s12864-015-2213-x

**Published:** 2015-11-26

**Authors:** Roei Levy, Richard F. Mott, Fuad A. Iraqi, Yankel Gabet

**Affiliations:** Department of Anatomy and Anthropology, Sackler Faculty of Medicine, Tel Aviv University, Tel Aviv, Israel; Wellcome Trust Centre for Human Genetics, University of Oxford, Oxford, UK; Department of Clinical Microbiology and Immunology, Sackler Faculty of Medicine, Tel Aviv University, Tel Aviv, Israel

**Keywords:** Trabecular bone microarchitecture, Collaborative cross, Osteoporosis, Fracture risk, Genome-wide association study, QTL mapping, Mouse genetics

## Abstract

**Background:**

The microstructure of trabecular bone is a composite trait governed by a complex interaction of multiple genetic determinants. Identifying these genetic factors should significantly improve our ability to predict of osteoporosis and its associated risks. Genetic mapping using collaborative cross mice (CC), a genetically diverse recombinant inbred mouse reference panel, offers a powerful tool to identify causal loci at a resolution under one mega base-pairs, with a relatively small cohort size.

Here, we utilized 31 CC lines (160 mice of both sexes in total) to perform genome-wide haplotype mapping across 77,808 single-nucleotide polymorphisms (SNPs). Haplotype scans were refined by imputation with the catalogue of sequence variation segregating in the CC to suggest potential candidate genes. Trabecular traits were obtained following microtomographic analysis, performed on 10-μm resolution scans of the femoral distal metaphysis. We measured the trabecular bone volume fraction (BV/TV), number (Tb.N), thickness (Tb.Th), and connectivity density (Conn.D).

**Results:**

Heritability of these traits ranged from 0.6 to 0.7. In addition there was a significant (*P* < 0.01) sex effect in all traits except Tb.Th. Our haplotype scans yielded six quantitative trait loci (QTL) at 1 % false discovery rate; BV/TV and Tb.Th produced two proximal loci each, on chromosome 2 and 7, respectively, and Tb.N and Conn.D yielded one locus on chromosomes 8 and 14, respectively. We identified candidate genes with previously-reported functions in bone biology, and implicated unexpected genes whose function in bone biology has yet to be assigned. Based on the literature, among the genes that ranked particularly high in our analyses (*P* < 10^-6^) and which have a validated causal role in skeletal biology, are *A*vp, *Oxt*, *B2m* (associated with BV/TV), *Cnot7* (with Tb.N), *Pcsk6*, *Rgma* (with Tb.Th), *Rb1,* and *Cpb2* (with Conn.D). Other candidate genes strongly suggested by our analyses are *Sgcz*, *Fgf20* (associated with Tb.N), and *Chd2* (with Tb.Th).

**Conclusion:**

We have demonstrated for the first time genome-wide significant association between several genetic loci and trabecular microstructural parameters for genes with previously reported experimental observations, as well as proposing a role for new candidate genes with no previously characterized skeletal function.

**Electronic supplementary material:**

The online version of this article (doi:10.1186/s12864-015-2213-x) contains supplementary material, which is available to authorized users.

## Background

Osteoporosis is the most common bone disease, affecting nearly half the US population over the age of 50 years, and is expected to cost over $25.3 billion by 2025 [1] per-annum. Worldwide, the incidence of bone fractures is expected to increase by 2.7-fold by 2050, due to population aging [2]. Importantly, women have a 1.5-fold higher risk of bone fracture than men throughout their lives as well as a 1.3-fold higher risk of hip or vertebral fracture than their risk of breast cancer [3–5]. A number of studies have shown that peak bone mass is tightly affected by host genetic determinants, and low bone mass significantly increases the risk of osteoporosis, especially in the elderly [6]. Risk of fracture is determined largely by the combination of bone structural parameters associated with bone strength. Most of these parameters, e.g. trabecular bone density, achieve peak values at skeletal maturity and subsequently decrease due to aging and menopause [6]. Microstructural features of trabecular bones, as those of cortical bones and indeed of most phenotypes, are known to be complex rather than Mendelian traits, determined by the cumulative effects and interactions of numerous genetic loci and environmental factors [7, 8].

Individuals with the same bone mineral density (BMD) measured by two dimensional dual energy x-ray absorptiometry (DXA) scans have different risks for fracture, suggesting that factors other than density, such as microstructural architecture, are important determinants of skeletal strength [9]. Recently, a number of genome-wide association studies (GWAS), including a meta-analysis, described >50 loci associated with bone mineral density (BMD) in humans [10–15]. However, many candidate genes, such as vasopressin (*Avp*), oxytocin (*Oxt*), and β*-*2-microglobulin (*B2m*) were not confirmed by GWAS, despite their proven role in bone metabolism [12, 13, 15–18]. Failure to significantly associate these genes to bone density in GWAS suggests that there may be other bone phenotypes not yet studied [17], or that genetic variation segregating in the populations tested does not influence the expression of these genes appreciably.

Importantly, almost all previous GWAS have used areal bone mineral density (aBMD) as the sole parameter for the bone phenotype. Clinically, areal and volumetric BMD (aBMD and vBMD) do not accurately predict risk fracture, suggesting that site-specific changes at the microstructural level are important determinants of bone strength. BMD as measured by DXA is a two dimensional projection that cannot determine or account for bone size, individual bone compartment shape (trabecular *vs* cortical), or the underlying microstructure. Notably, fracture risk and bone strength are tightly associated with changes in the bone microstructure, but are not always detected by DXA and peripheral computed tomography (pQCT) [19]. There is growing evidence that cortical and trabecular bone have distinct genetic influences and should be analyzed separately [15, 16]. Indeed, a recent GWAS in collaborative cross (CC) mice, based on DXA measurements, failed to reveal any heritability of BMD [20], whereas our preliminary analyses in the same mouse panel (some of these data are as yet unpublished, others are reported herein) show highly significant heritability levels in most of the microstructural parameters measured by micro-computed tomography (μCT).

The CC is a panel of recombinant inbred lines of mice descended from eight genetically divergent strains [21], designed for high resolution analysis of complex traits, with particular emphasis on traits relevant to human health [22, 23]. In this study we used 31 CC lines which at the time of the experiment were over 90 % homozygous. GWAS in mice traditionally use rodent inbred line crosses with poor mapping resolution of 20 to 40 Mb [24], thus proposing hundreds of genes as potential candidates. To narrow the widths of QTLs, combined cross-analysis and block haplotyping techniques have been proposed [24, 25]. Mapping resolution in the CC is typically of the order of 1–2 Mb, due to the increased extent of observable recombination in the population and the use of haplotype-based tests of association [18, 22–25]. Genetic variation segregating in the CC is much greater (over 30 million SNPs, due to the inclusion of three wild-derived strains) than in a traditional intercross between standard laboratory strains (about 4 million SNPs) [7]. In addition to the narrower QTLs typically generated using the CC panels, this allows the identification of a greater number of contributing genetic variants as compared to panels based on classical strains only. Moreover, by using the extensive catalogues of variation in the founders (see Keane et al., 2011 [7]) it is possible to impute the variants into each CC lines and test for association [26]. By merging the CC founders according to the strain distribution pattern (SDP) of their alleles at potential quantitative trait nucleotides (QTN) within a given QTL (obtained by the initial haplotype-based mapping) [26, 27], one can refine the initial haplotype mapping and identify candidate genes.

Here, we aimed to identify new genetic determinants of bone microarchitecture and metabolism associated with bone strength in CC mice. In contrast to working with human populations in which the environment is uncontrolled and individuals are genetically distinct, in the CC we could collect phenotypes in a controlled environment and from multiple individuals with the same genetic background. We measured microarchitectural trabecular bone traits, including trabecular bone volume fraction (BV/TV), trabecular bone number (Tb.N), trabecular bone thickness (Tb.Th), and trabecular bone connectivity (Conn.D) in the distal femoral metaphysis. Our results provide the first genome-wide confirmation of the involvement of several genetic factors in trabecular bone development as well as suggest new candidate genes with undocumented roles in bone metabolism.

## Results

### Trabecular traits vary across the CC population

In each CC animal we analyzed the trabecular bone compartment of the distal femoral metaphysis and measured trabecular bone volume fraction (BV/TV; range = 2.38 – 29.2 %), trabecular number (Tb.N; range = 0.63 – 5.64 mm^-1^), trabecular thickness (Tb.Th; range = 35.95 – 60.0 μm), and trabecular connectivity (Conn.D; range = 14.0 – 205.09 mm^-3^). Heritability (H^2^) for all traits was greater than 0.6, i.e., genetic differences between the lines explained most of the phenotypic variation (Table 1). There was a high phenotypic heterogeneity between lines for all traits except for Tb.Th. While phenotypic heterogeneity was observed in both males and females, female variability among the lines was greater (Additional file 1: Figure S1).

**Table 1:** Heritability, sex, and age effects for trabecular traits

Trait	H^2^	logP	Sex effect	Sex logP	Age effect	Age logP
BV/TV	0.63	15.00	0.65	2.30	0.57	3.80
Tb.N	0.71	15.00	0.72	5.00	-	-
Tb.Th	0.56	11.00	0.56	0.04	0.01	15.00
Conn.D	0.67	15.00	0.70	8.58	-	-

The Heritability (H^2^), sex, and age effects and their respective significance (given as log*P* = -log_10_(*P* value)). Note that except for BV/TV, age effect is negligible, although the statistic is high for Tb.Th; this is due to the low variability of the age when compared to the high variability of Tb.Th. When values for both age effect and its respective *P*-value are absent both were low

**Fig. 1:**
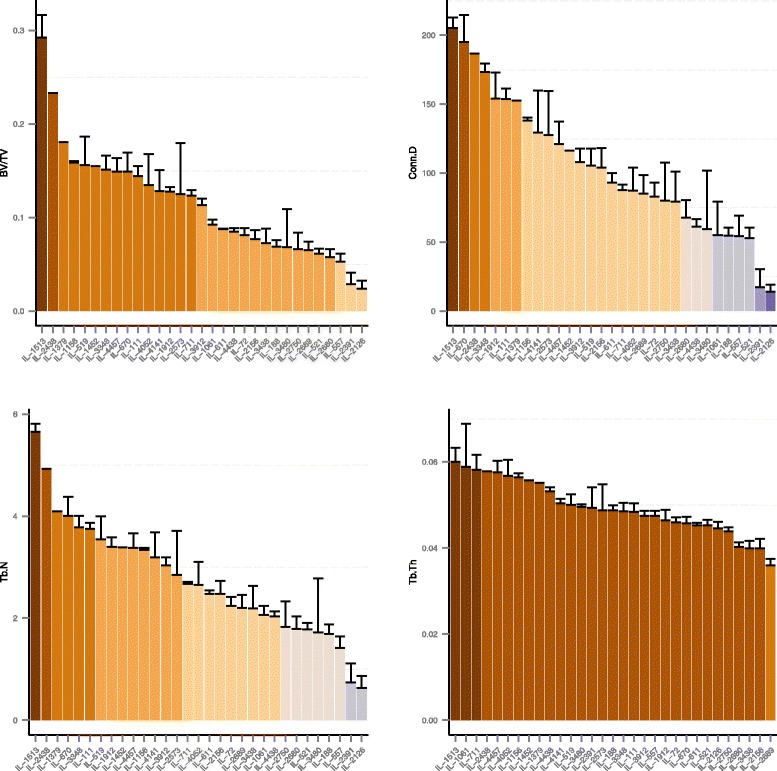
Mean trabecular values obtained from 31 CC lines. From top-left, counter-clockwise: trabecular bone volume fraction (BV/TV; %), trabecular number (Tb.N; mm^-1^), thickness (Tb.Th; mm), and connectivity density (Conn.D; mm^-3^). Lines were grouped according to Duncan’s LSR; each color-coded group is statistically different from all the other groups (*P* < 0.001; Tukey’s ANOVA-based test for multiple comparisons). Bars are Mean + SE

**Fig. 2:**
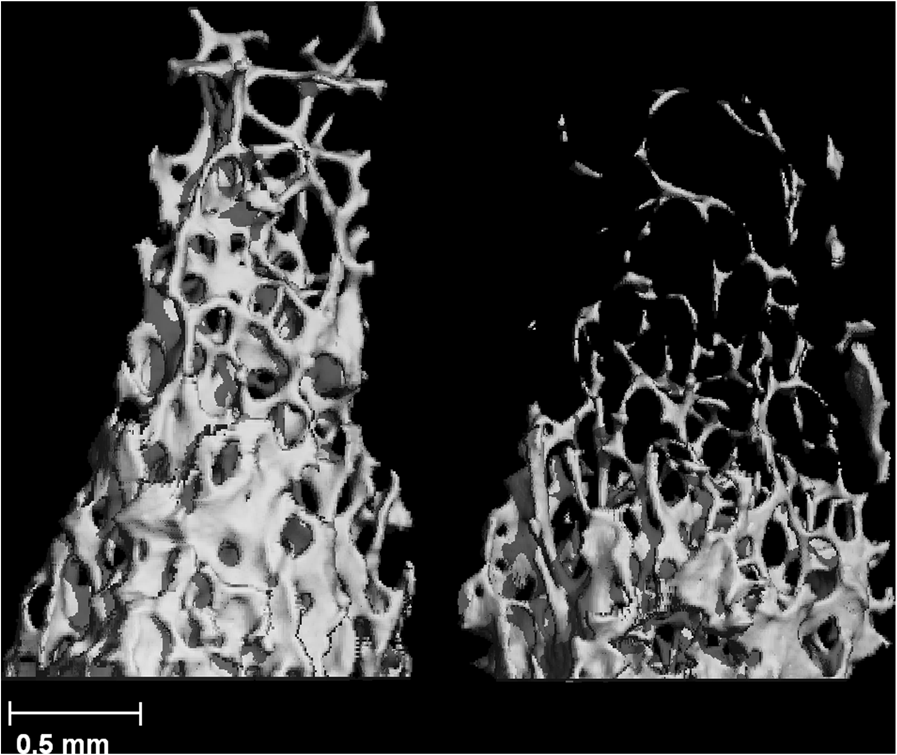
Two representative μCT images of the femoral trabecular compartment. Left, IL-1513. Right, IL-2689. Note the structural differences due solely to the genetic background

Figure 1 shows the phenotypic distribution across lines, sorted in descending order by their mean. Lines are color-coded according to Duncan’s least significance range (LSR). This partitioned the lines to at least three groups of lines in which each group significantly differs from the others (*P* < 0.001). Additional file 2: Table S1 lists the means of the lines for each trait. To emphasize the phenotype variations across the lines, we identified the two lines with extreme values for BV/TV, Tb.N, and Conn.D (Figs. 1 and 2). Interestingly, in all traits except Tb.Th, IL-1513 was at the upper-most extreme and IL-2126 and IL-2391 (and in BV/TV also IL-557) were at the lower-most extreme (Figs. 1 and 2).

Our analyses showed that all phenotypes were approximately normally distributed and positively correlated with one another (Additional file 2: Table S2). Pearson’s correlation coefficient (*r*) ranged between 0.93 for BV/TV *vs*. Tb.N, and 0.29 for BV/TV *vs*. Tb.Th. However, despite these overall high phenotypic correlations, some lines were discordant between the bone traits. For example, IL-670 had high trabecular Conn.D (88.82 mm^-3^) but average Tb.N and BV/TV (2.42 mm^-1^ and 8.8 %), and low Tb.Th (45.73 μm). IL-557 was in the lower range for BV/TV, Tb.N and Conn.D (5.3 %, 1.42 mm^-1^ and 47.86 mm^-3^) but was average for Tb.Th (47.43 μm).

**Fig. 3:**
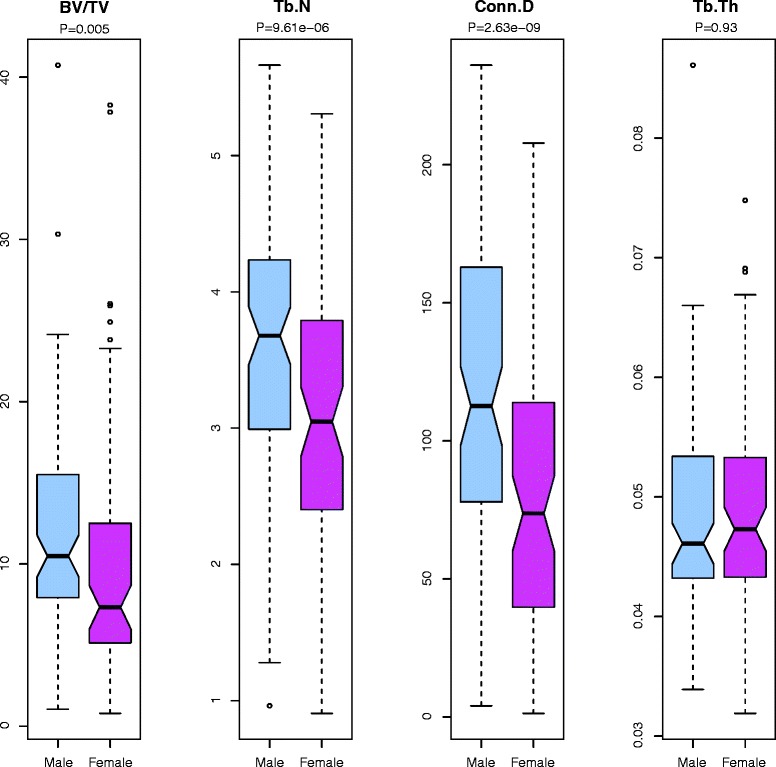
Sex effects on trabecular traits. Notched box-and-whisker plots demonstrating the interquartile range (IQR; median in bold, whiskers correspond to ± 1.5 · IQR) for BV/TV (%), Tb.N (mm^-1^), Tb.Th (mm), and Conn.D (mm^-3^). Significance is reported in logP values (a negative log-transform of the *P*-value, to emphasize the extent to which they differ). Note that except for Tb.Th, all traits had a significant sex effect (*P* < 0.01, or logP > 2)

Because body weight is often correlated with bone density [28, 29], we assessed the effect of weight on our examined traits. Irrespective of sex effects, our data indicate that body weight did not affect any of the analyzed bone parameters in this animal sample (Pearson’s *r* ≤ 0.11). In contrast, sex had a significant impact on BV/TV, Tb.N, and Conn.D (*P* < 0.001), but not on Tb.Th (Table 1, Fig. 3). Notably, males always averaged higher for the three traits where we observed a sex effect (Fig. 3 and Additional file 1: Figure S1). Although age was in the limited range of 10–13 weeks, we did observe a significant age effect for BV/TV (*P* < 0.001, Table 1).

### Mapping the albino trait supports a modest number of CC lines

As a positive control, we performed a QTL mapping for albinism using the same mice from 31 CC lines in which we measured the bone traits, and in an additional 7 lines in which no skeletal analysis was performed (38 lines in all). Using the same methodology, we were able to map albinism to its known locus on chromosome 7 [30]. This confirms that the identities of the phenotyped lines matched their expected genotypes and provides validation for using a small number of CC lines for QTL mapping (Additional file 3: Figure S2).

### Haplotype association mapping reveals six QTLs

**Fig. 4:**
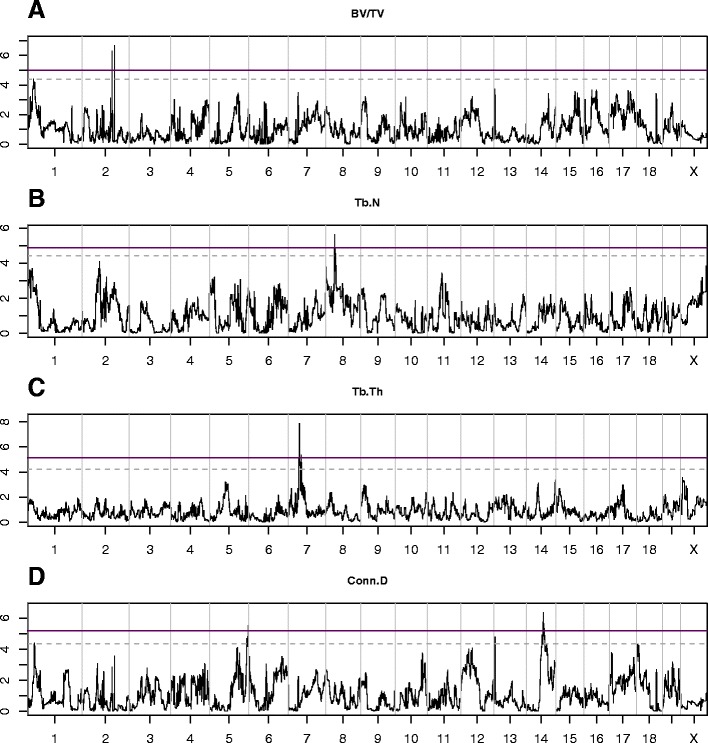
QTL haplotype mapping of trabecular traits. Plots show –logP values according to the chromosome location (x-axis). Horizontal line represent the 99^th^ (solid) and 95^th^ (dashed) percentile thresholds for genome-wide significance. **a** The scan for BV/TV yielded two proximal QTLs, at chromosome 2, with their peaks at a -logP of 6.67 and 6.31, respectively. **b** Tb.N had a QTL at chromosome 8 with a -logP of 5.63. **c** Tb.Th had two QTLs at chromosome 7 with a -logPs of 7.87 and 5.36. **d** Conn.D had a QTL at chromosome 14 with a -logP of 6.36

After adjusting for sex and age and taking the mean for each line, we performed a haplotype association analysis (Fig. 4). Using the full database for each trait, we obtained haplotype mappings which were correlated in a manner that echoed the phenotype correlation; this practice has limitations in that it can mask important loci. In other words, the complex nature of the trabecular traits is prone to loss of information owing to presumed complementary genetic factors that govern each trait. This phenomenon dilutes the sample and adds noise which accumulates false negatives. QTL masking can also be due to genetic drift occurring in inbred population of high generation [31, 32]. To overcome this hindrance we sampled subsets from our cohort and unlocked otherwise confounded loci (this was achieved with a minimum of 28 lines); here, therefore, each trait is founded on the set that maximizes its loci mapping’s peaks. For BV/TV and Tb.Th, we found two proximal QTLs for each on chromosome 2 and 7, respectively. Tb.N and Conn.D yielded one QTL on chromosome 8 and another on chromosome 14, respectively. The two QTLs for BV/TV, the QTL for Tb.N, the two QTLs for Tb.Th, and the QTL for Conn.D, are hereafter referred as trabecular-related loci (*Trl)* 1 to 6, respectively.

All QTLs are above the genome-wide 99^th^ percentile threshold; they were obtained empirically by 200 permutation simulations, with an experiment-wide false discovery rate (FDR) under 1 % for each scan. As shown in Table 2 and Additional file 4: Figure S3, the respective widths of the 50 % confidence intervals (CIs) for *Trl*1 to 6 were 0.2, 0.3, 3.27, 0.2, 0.13, and 9.94 Mb. We also calculated 50 % CIs according to Li’s method (Table 2, [33]). The CIs obtained by our simulations were comparable to those calculated after Li, albeit the latter were usually slightly wider.

**Table 2:** Positions of QTLs associated with trabecular traits

QTL	Trait	Chr	logP	99th % threshold logP	Sig	H^2^_r_	logP H^2^_r_	50 % CI				90 % CI		95 % CI	
								Simulation		Li (2011)					
								Position	Width (Mb)	Position	Width (Mb)	Position	Width (Mb)	Position	Width (Mb)
*Trl*1	BV/TV	2.00	6.67	5.01	0.01	0.60	2.46	130.48-130.68	0.20	129.9-130.5	0.60	128.08-132.8	4.70	126.2-135.6	9.40
*Trl*2	BV/TV	2.00	6.31	5.01	0.01	0.67	3.37	121.9-122.2	0.30	119.6-123.4	3.80	121.7-122.4	0.70	115.6-124.9	9.30
*Trl*3	Tb.N	8.00	5.63	4.88	0.01	0.61	2.42	37.2-40.47	3.27	37.8-41.2	3.40	31.8-43.9	12.40	30.8-46.9	16.10
*Trl*4	Tb.Th	7.00	7.87	5.14	0.01	0.86	6.75	64.6-64.8	0.20	63.7-65.6	1.90	63.8-65.7	1.90	62.7-68.1	5.40
*Trl*5	Tb.Th	7.00	5.36	5.14	0.01	0.73	12.77	73.4-73.5	0.13	73.2-73.7	0.52	70.4-75.1	4.74	66.01-77.9	10.73
*Trl*6	Conn.D	14.00	6.36	5.17	0.01	0.66	12.77	69.8-79.74	9.94	70.7-79.2	8.50	66.7-77.2	10.62	64.7-77.1	13.50

*Chr* chromosome; logP = negative 10-base logarithm of *P* value; Sig = genome-wide significance level; 99th % threshold logP = threshold used to define cut-off for QTL peaks (Fig. 4); H_r_^2^ = regional heritability. Positions and widths of the simulation-based 50, 90, and 95 % CIs are given, as well as 50 % CIs calculated after Li (2011). (See text.)

We then estimated the regional heritability (H^2^_r_) explained by each QTL and found the combined values were approximately equal to the genome-wide H^2^. For the 6 defined *Trl*, the H^2^_r_ range fell between 0.60 and 0.86 (Table 2).

**Fig. 5:**
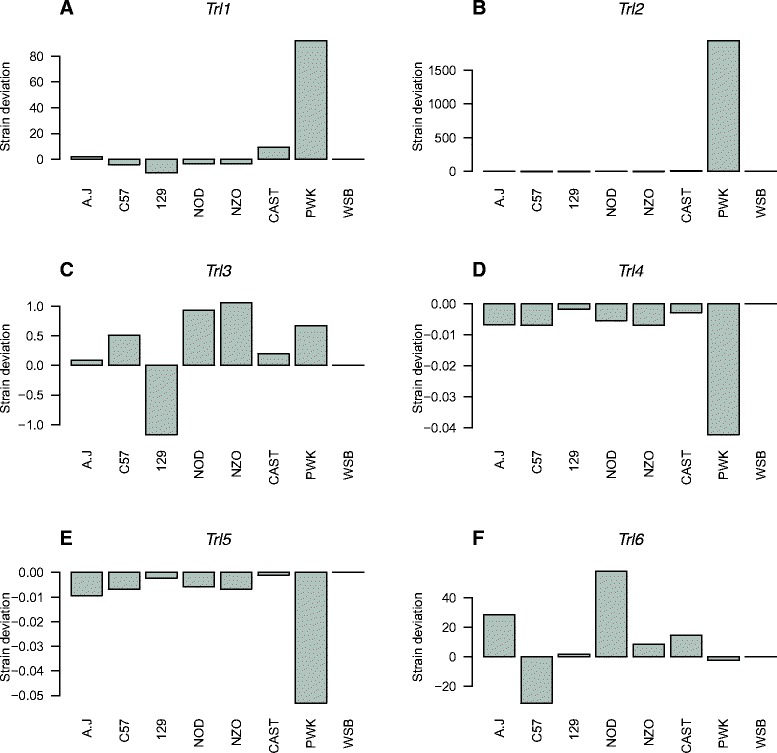
Founder effects at each QTL. Each panel corresponds to one of the six mapped trabecular-related loci (*Trl*). The x-axis shows the eight founders of the CC population and the y-axis shows the haplotype effects predicted from a regression fit to the founder haplotype probabilities at the QTL. All effects are plotted relative to WSB which is arbitrarily set to 0. **a** Trl1 (BV/TV). **b** Trl2 (BV/TV). **c** Trl3 (Tb.N). **d** Trl4 (Tb.Th). **e** Trl5 (Tb.Th). **f** Trl6 (Conn.D)

Subsequently, we assessed the haplotype contribution of the founder strains to each QTL (Fig. 5); *Trl*1, *Trl*2, *Trl*4, and *Trl*5 associated with BV/TV and Tb.Th, were predominantly affected by the PWK founder strain. In contrast, the loci associated with Tb.N and Conn.D (*Trl*3 and *Trl*5, respectively) had a more varied founder contribution.

### Imputation identifies candidate genes

**Fig. 6:**
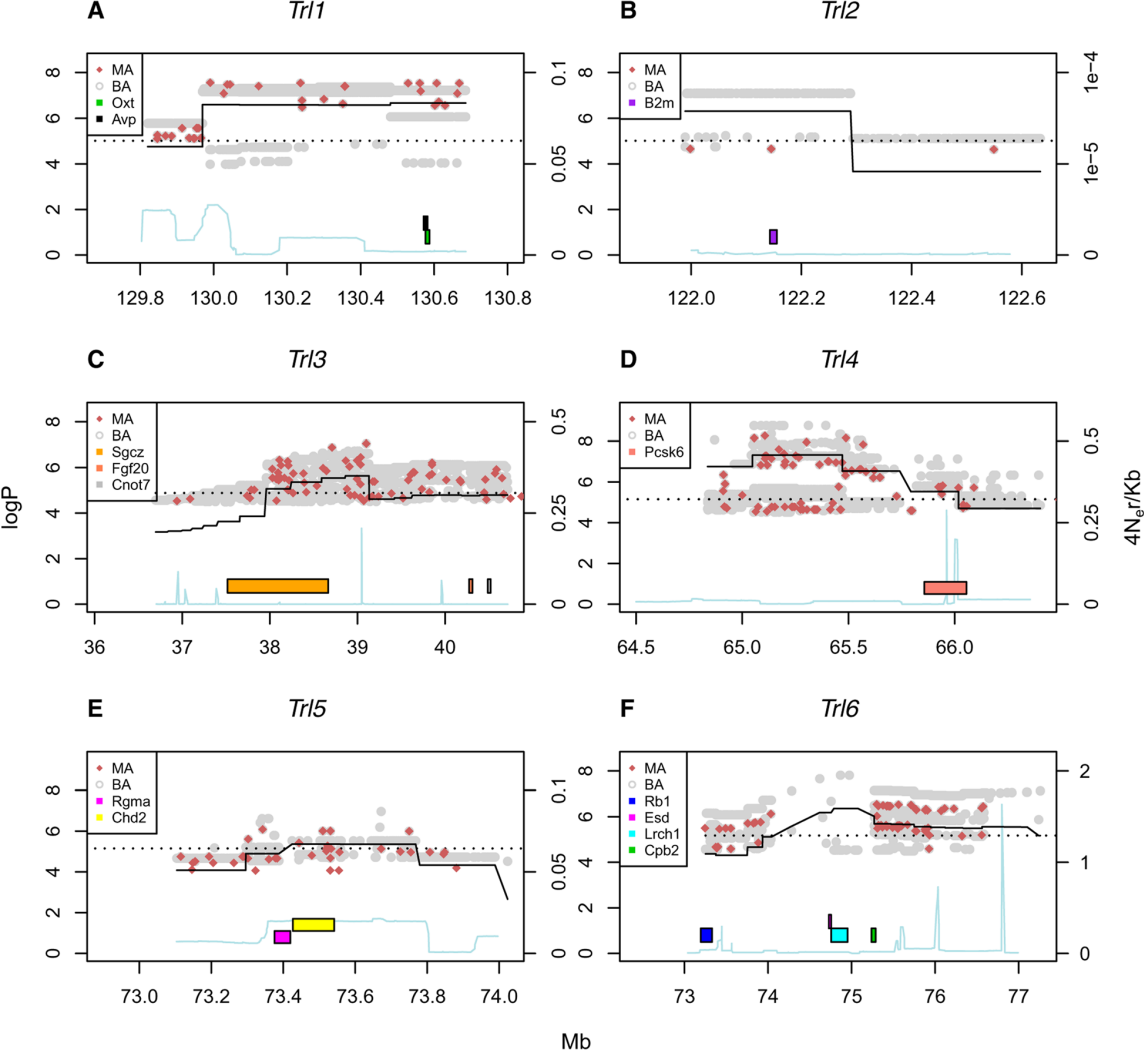
Merge analysis at each QTL. Each panel corresponds to one of the six mapped QTLs. The x-axis is the genome location; the left y-axis is the logP of the association test between the trait and the locus; and the right y-axis is the recombination rate scale, in 4N_e_r/Kb. The continuous black line is a section of the haplotype-based genome scans in Fig. 4. The red (MA; multiallelic) and gray (BA; biallelic) dots correspond to the results of the merge analyses. Genes appear in the legend as colored bars from top to bottom as their order of appearance in the image, from left to right. Dotted line is the 99^th^ percentile threshold from Fig. 4. Blue continues line represents recombination rates. For clarity, merge logP values below 4 are not shown. **a** *Trl*1 (BV/TV). **b** *Trl*2 (BV/TV). **c** *Trl*3 (Tb.N). **d** *Trl*4 (Tb.Th). **e** *Trl*5 (Tb.Th). **f** *Trl*6 (Conn.D)

To pinpoint candidate genes we fine-tuned our model using merge analysis (Fig. 6, [26]) to test the association of variants imputed from the catalogue of variation in the eight CC founders. When the logP value of the imputation test is lower than that of the haplotype analysis, the SNPs at the QTL peak are unlikely to be causal; in contrast when the merge logP is higher, it suggests that the tested variant is consistent with being a quantitative trait nucleotide (QTN), with the caveat that there may be multiple variants with the same SDP that are equally likely to be causal [34]. Filtering out SNPs with logP values lower than that of the haplotype-scan peak SNPs we were able to identify candidate genes.

The list of all potential causal variants identified by the merge analysis is presented in Additional file 2: Table S3. In accordance with the relatively wide 50 % CI of Conn.D (*Trl*6), the peak of the haplotype analysis did not contain the highest merge logP values; rather, these lay within ±3 Mb from the inferred peak. Regarding *Trl*4 and *Trl*5 (Tb.Th), their 50 % CI is much narrower than that of *Trl*6 (0.2 and 0.13 vs 9.94 Mb, respectively, Table 2 and Additional file 4: Figure S3), but the peak of the QTL lacks a high density of SNPs with merge logP higher than the haplotype-scan logP threshold (Fig. 6). Moreover, those present are multiallelic, a situation which reflects the complexity observed in the predicted founders’ haplotype effects, and suggesting the presence of multiple causal variants (Fig. 6). To gain further insight into the *Trl*s, we included recombination rates for the mouse at 4N_e_r/Kb, following Brunschwig et al. [35] (Fig. 6); although most loci are in regions of mild recombination rates, data for *Trl*3*,* 4, and 6 suggest that these loci are in the vicinity of recombination hot spots. We note that this did not alter the imputation performance (see Discussion).

We identified 11 potential genes for the six *Trls*; we found three genes for BV/TV (*Trl*1 and *Trl*2), three for Tb.N (*Trl*3), three for Tb.Th (*Trl*4 and *Trl*5), and two for Conn.D (*Trl*6). Importantly, our analysis revealed genes that already have well-documented functions in bone biology as well as genes that have yet an unexplored role in the regulation of trabecular bone microarchitecture (Additional file 2: Table S3).

## Discussion

To our knowledge, this study is the first to use the CC population in a genome-wide association analysis of high-resolution trabecular bone traits. Our results replicate previous association analyses, confirm previous in vivo and in vitro experiments, and suggest new candidate genes for the regulation of trabecular bone microstructure. The roles of these latter genes will require validation in future studies both in vivo and in vitro. We thus provide a valuable resource for further research aimed at understanding the underlying genetic determinants of complex trabecular structure.

Our association analyses were enhanced by virtue of ancestral sequence information that enabled us to deduce QTNs that segregate differentially in the CC lines, thus reducing false positives and assisting in the identification of potential candidate genes. This imputation’s power depends on (a) the accuracy of the catalogues of variants in the CC founders (as determined from resequencing the strains by Keane et al. 2011 [7]) and (b) the accuracy with which the genome mosaics of the CC lines are determined from SNP genotypes using the HAPPY R package. Whilst we cannot control for sequencing errors in the founders, any uncertainty in the locations of mosaic breakpoints is encoded in the probability distribution of descent from each of the eight founders. At loci with high certainty (which are the great majority) the probability distribution is concentrated on one founder which has a much higher probability (close to 1) than that other strains (all close to 0). Around breakpoints the probability distribution transitions through a short region of uncertainty in which the strain probabilities are more equal. The way imputation (merge analysis) is performed in the CC is to compute the dosage of a SNP allele in a CC line by adding together the probabilities for those founders carrying that allele in the variant catalogue. This means that in regions of low certainty (where the founders are equally likely) the dosage of the allele will be approximately proportional to its frequency among the founders. In regions of high certainty the dosage is dominated by the founder strain with highest probability (i.e., the dosage will be close to 0 or 1). Genetic association with the SNP is determined by linear regression on the imputed dosages. The effect on association testing is that at a given SNP, the majority of CC lines will have high-confidence dosages close to 0 or 1, and a small minority (those with breakpoints near the SNP) will have intermediate dosages. These latter have relatively little impact on the statistical significance of the association, which is driven by the high-confidence lines.

We were able to narrow the interval to the kilo-base range by coinciding the CI with the 99^th^ percentile threshold and imputation results for four out of six QTLs, namely*, Trl*1, *Trl*2 (for BV/TV), *Trl*4*,* and *Trl*5 (for Tb.Th), and thus to focus on a more restricted number of candidate genes.

Our findings indicate 4 *Trls* for which there was a strong founder effect by PWK. This emphasizes the advantage of the CC mouse panel, inasmuch as it includes 3 wild-derived strains, including the PWK strain. This strain differs from classical laboratory strains by about 17 million SNPs [7], and it is likely to carry rare variants not found in the classical inbred strains. This unique allelic diversity results in the presence of variants in more genes than in inbred populations, thus enabling the detection of a larger array of genes that contribute to the analyzed traits.

We detected 3 genes associated with BV/TV; two under *Trl*1 and one under *Trl*2. Among these, *Oxt* encodes a precursor of oxytocin, and *Avp* encodes arginine-vasopressin. Both of these proteins are hormones produced in the hypothalamus and secreted from the posterior pituitary gland, and differ from each other by only two amino acids. Tamma et al. have previously shown that these hormones have a direct skeletal effect – affecting BV/TV, Tb.N, and Tb.Th - in both males and females. They act via the hypothalamus-pituitary-skeletal axis [12, 13]. *Oxt* affects both osteoblasts and osteoclasts; it increases osteoblast differentiation by upregulating bone morphogenic protein (BMP) 2, and stimulates osteoclasts directly via activation of NF-kB and MAP kinase signaling, and indirectly via increasing RANK-L expression. Concordantly, *Oxt*-null mice exhibit an osteoporosis phenotype [12]. *Avp* deficiency, similarly, renders mice susceptible to bone loss via activation of the *Avpr1a* and *Avpr2* G-protein coupled receptors, present in both osteoclasts and osteoblasts, which in turn activate the Erk pathway [13]. *B2m* encodes β2-microglobulin, which is involved in the regulation of the host immune response and is known to activate osteoclasts and osteoblasts in both sexes. It supports the transition of epithelial to mesenchymal cells, thereby promotes bone metastases originating from prostate, breast, lung, and renal cancers [18]. It does so mainly by interacting with hemochromatosis (HFE) protein. HFE null mice were found to be phenotypically identical to BMP2-null mice [36]. Further, men with a mutation in HFE had significantly lower BMD [37]. Interestingly, a recent study found that lysyl oxidase (LOX) plays a major role in the facilitation of bone metastases [38]; BMP1 proteolytically cleaves Pro-LOX to active LOX [39].

Under the QTL for Tb.N we identified *Cnot7*, *Fgf20,* and *Sgcz* to be the most prominent contributors to the phenotype. *Cnot7* - or CCR4-NOT transcription complex, subunit 7 - suppresses bone mass and inhibits BMPs in osteoblasts [40]; Cnot7-null male mice had a substantial increase in bone mass, supposedly by inhibiting the expression of alkaline phosphatase induced by BMPs in osteoblastic cells. *Fgf20* and *Sgcz* (fibroblast growth factor and ζ-sarcoglycan, respectively) have no documented role in bone metabolism. *Fgf20* is a transcriptional target of β-catenin which is important during Wnt signaling [41] and interacts with BMP7 [42]. Wnt/β-catenin signaling is an established pathway for normal bone homeostasis [43]. *Sgcz* is expressed in both striated and smooth muscles. Importantly, bone and muscle share some common genes [44]. As of yet, it remains unclear whether these proteins affect both sexes equally and thus further work is needed to examine their differential expression in males and females.

For Tb.Th the strongest candidate genes were *Pcsk6*, *Rgma,* and *Chd2. Pcsk6* encodes paired amino acid converting enzyme 4 (PACE4) which, in human females, evokes proteolytic degradation of aggrecan, a major cartilage molecule, by activating the proteases ADAMTS-4 and pro-ADAMTS-4, thereby inducing the development of osteoarthritis [45]. Interestingly, tibial trabecular bone structure was found to be a good predictor of knee osteoarthritis [46]. *Rgma* (encodes repulsive guidance molecule A) has been shown to be a co-receptor of BMP-2 and BMP-4, by binding directly to these factors, and to mediate their signaling via BMP type I and II receptors [36, 47, 48]. Based on the International Mouse Phenotyping Consortium database [49], both female and male mice with mutant *Chd2* (encodes chromodomain helicase DNA binding protein 2) have a profoundly higher bone mineral content and greater bone area, although these findings have yet to be published in a peer-reviewed journal.

We speculate that putative skeletal role for genes associated with Conn.D can be assigned to *Cpb2*, *Rb1*, and *Esd. Cpb2* (carboxypeptidase B2) has been implied to confer a protective role in the pathogenesis of osteoarthritis in male mice [50]. *Rb1* was found to have a role in osteogenesis [51]. Again, further work is needed to determine if this role is sex-dependent.

Importantly, the peak of the QTL for Conn.D had almost no significant merge logP SNPs and the 50 % CI was large enough to speculate that genes therein are false positives; there reside *Lrch1* (encodes Leucine-Rich Repeats And Calponin Homology (CH) Domain Containing 1) and *Esd* (encodes esterase D). *Lrch1* was found in a previous GWAS exploring the knee osteoarthritis phenotype in humans [52], but large-scale replication studies [53, 54] then dismissed it from being casual. However, *Esd* was confirmed by microarray-based qPCR analysis to influence spinal bone mineral density in inbred rats [8] and thus seems to be a likely candidate. Still, due to insufficient evidence in our and previous studies, the role of these genes in bone microstructure remains unclear.

The genes aforementioned are most likely to explain the *Trls*, based on published functionality assays. However, it is possible that other genes in the vicinity of these loci are related to the traits examined. While these cannot be ruled out conclusively without carrying out particular validation experiments, they are unlikely to play a prominent role in determining the trabecular microstructure given their known molecular role. Of note, considering the meaningful sex and age effect some of the phenotypes exhibit, we expect some genes to have a sex and age differential behavior, even though none of the loci (and their human orthologues) were found on either of the sex chromosomes (Additional file 2: Table S3). Lastly, besides *Avp* and *Oxt* which are confirmed to affect the microstructural parameters we studied here, others have yet to be validated for these skeletal traits.

## Conclusions

The variation of trabecular phenotypes between perfectly viable CC lines reflects in many aspects the variance in human populations. Whilst the complete complex spatial and temporal interactions of the genes controlling trabecular traits cannot be expected to be identified with a limited cohort size at our disposal, nevertheless, using a relatively small cohort with sufficient replication in each line, the wide phenotypic range of divergence enabled us to dissect the genetic architecture of trabecular phenotypes, and to direct previous genes associated with bone to more refined trabecular microstructural parameters.

## Methods

### Mice

Mice aged 10 to 13 weeks (male *n* = 86; female *n* = 74), from 31 different CC lines (average of 5 mice per line) were used in this study. The mice were at inbreeding generations of 11 to 37, which correspond to 80–99.9 % genetic homozygosity, respectively. The mice were bred and maintained at the small animal facility of the Sackler Faculty of Medicine, Tel Aviv University (TAU), Israel. They were housed on hardwood chip bedding in open-top cages, with food and distilled water available *ad libitum*, in an identical controlled environment (temperature = 25 ± 2 °C; 60 % ≤ humidity ≤ 85 %) and a 12 h light/dark cycle. All experiments protocols were approved by the Institutional Animal Care and Use Committee (IACUC M-13–014) at TAU, which follows the NIH/USA animal care and use protocols.

### Specimen collection

Mice were intraperitoneally euthanized with cervical dislocation performed approximately one minute after breathing stops owing to 5 % Isoflurane inhalation. Left femora were harvested and fixed for 24 h in 4 % paraformaldehyde solution, and then stored in 70 % ethyl alcohol.

### μCT evaluation

Whole left femora from each mouse were examined as described previously [55] by a μCT system (μCT 50, Scanco Medical AG, Switzerland). Briefly, scans were performed at a 10-μm resolution in all three spatial dimensions. The mineralized tissues were differentially segmented by a global thresholding procedure [56]. All morphometric parameters were determined by a direct 3D approach [57]. Parameters analyzed were determined in the metaphyseal trabecular bone, which included trabecular bone volume fraction (BV/TV), trabecular thickness (Tb.Th), trabecular number (Tb.N), and trabecular connectivity density (Conn.D).

### Genotyping

A representative male mouse from each line was initially genotyped with a high mouse diversity array (MDA), which consists of 620,000 SNPs (Durrant et al., 2011). After about two intervals of 4 generations of inbreeding, all the CC lines were regenotyped by mouse universal genotype array (MUGA, 7,500 markers) and finally with the MegaMuga (77,800 markers) SNP array to confirm their genotype status [21]. The founder-based mosaic of each CC line was reconstructed using a hidden Markov model in which the hidden states are the founder haplotypes and the observed states are the CC lines, to produce a probability matrix of descent from each founder. This matrix was then pruned to about 11,000 SNPs by averaging across a window of 20 consecutive markers for faster analyses and reduction of genotyping errors [58].

### Statistical analyses

All statistical analyses were performed with the statistical software R (R core development team 2009), including the package happy.hbrem [59].

#### Heritability and sex and age effects

Broad-sense heritability (H^2^) was obtained for each trait by fitting the trait (the independent variable) to the CC line label in a linear regression model that incorporates sex and age. ANOVA test was used to compare a null model (in which all dependent variables are set to 0) with linear models that fit the sex, age and the CC line labels to the examined trait. Practically, the difference between the residual sum of squares (RSS; ${\displaystyle {\sum}_1^n{\left({\mu}_i - {\widehat{\mu}}_i\right)}^2}$) of the covariates model and that of the CC-line labels can be seen as the net genetic contribution to the trait. Thus, this difference divided by that of the covariate model gives an estimation of the heritability. Sex and age effects were calculated separately, by dividing the RSS difference between the null and full model with that of the null model. Let *F*_0_ be the model that fits the trait to the covariates; *F*_1_ the model that fits the trait to the covariates and the CC line label; and *F*_00_ the null model. Then, employing ANOVA, heritability is:

$$ {H}^2 = \left(RSS\left({F}_0\right)-RSS\left({F}_1\right)\right)\ /\ RSS\left({F}_{00}\right). $$

Similarly, sex and age effects are computed separately, by fitting separately each covariate in *F*_0_. The covariate effect is thus:

$$ \left(RSS\left({F}_{00}\right)-RSS\left({F}_1\right)\right)\ /\ RSS\left({F}_{00}\right). $$

#### Haplotype mapping

Each trait was fitted in a multiple linear regression model to the probability matrix of descent from each founder, including sex and age as covariates. The expected trait value from two ancestors, termed the genetic fit, is:

$$ {\mu}_i = \mu +{\displaystyle \sum_{s,t}}{F}_{Li}\left(s,t\right)\left({\beta}_s+{\beta}_t\right) = \mu +{\displaystyle \sum_s}{\displaystyle \sum_t}{F}_{Li}\left(s,t\right){\beta}_s $$

where *μ* is a normally distributed trait mean, with sex and age incorporated; *F*_*Li*_(*s*, *t*) is the probability of descent from founders *s* and *t*; and *β*_*s*_ + *β*_*t*_ is the additive effect of founders *s* and *t.* Because ${\displaystyle {\sum}_s}{\displaystyle {\sum}_t}{F}_{Li}\left(s,t\right)=2$ for a diploid organism, the maximum likelihood estimates ${\widehat{\beta}}_s$ are not independent. Thus, they are expressed here as differences from the WSB/EiJ founder effect, so that ${\widehat{\beta}}_{WSB}=0$. Number of members per line was weighted and integrated in the linear model. ANOVA was then used to compare this model with a null model where the founder effects are all set to 0; the resulting *F*-statistic yielded the significance of the genetic model *vs*. the null model and the negative 10-base logarithms of the P values (logP) were recorded.

Permutations of the CC lines between the phenotypes were used to set significance thresholds levels. Founder effects are the estimates derived from the multiple linear regression fit above.

Regional heritability (H_r_^2^) was hereafter computed by ANOVA as in the broad-sense heritability computation, except that here null linear regression fit was compared with a genetic linear regression fit with the probability matrix of the founder descent at the peak QTL as the explanatory variable.

False discovery rate (FDR) was calculated using the p.adjust function in R, with the method “BH”.

#### Confidence intervals

Confidence intervals (CIs) were obtained both by simulations and by the quick method of Li, 2011 [33]. In the simulations, we resampled the residuals of the original linear regression fit at the peak of each QTL and rescanned 100 intervals within 7–10 Mb of the original loci to find the highest logP. Accordingly, following Durrant et al. [27], 1000 QTLs were simulated: if ${\widehat{t}}_i$ is a random permutation of the residuals of fitted genetic model at the QTL peak, and *K* is a marker interval in a neighborhood of 3.5 to 5 Mb of the QTL peak *L*, a set of values for each trait, *Z*_*iK*_ is provided by:

$$ {Z}_{iK} = {\widehat{t}}_i \exp \kern0.5em \left(\widehat{\mu} + {\displaystyle \sum_s}{X}_{Kis}{\widehat{\beta}}_s\right). $$

#### Merge analysis

In the merge analysis the eight founder strains are partitioned and merged according to the strain distribution pattern (SDP) of the alleles at the quantitative trait nucleotides (QTN) within a given QTL (formerly obtained by the initial mapping). If we denote the polymorphism as *p,* then *X*_*p*_ = 1 if *s* has allele *a* at *p,* and *X*_*p*_ = 0 otherwise [34]. Then, at *p,* the probability of *i* to inherit alleles *a and b* from *s and t, respectively,* within *L* is

$$ {G}_{pi}\left(a,b\right) = {\displaystyle \sum_{s,t}}{X}_p\left(a,s\right){X}_p\left(b,t\right){F}_{Li}\left(s,t\right). $$

This merges the founder strains by *p.* The expected trait value in the merged strains can now be inferred by

$$ {\displaystyle \sum_{a,b}}{G}_{pi}\left(a,b\right)\left({\beta}_a+{\beta}_b\right). $$

Because this is a sub-model of the QTL model, it is expected to yield higher logP values due to a reduction in the degrees of freedom. Significance was obtained by comparing the merge model with the QTL model. Individual genes were extracted from the Sanger mouse SNP repository (http://www.sanger.ac.uk/sanger/Mouse_SnpViewer).

### Availability of supporting data

The data sets supporting the results of this article are available in the GitHub repository: https://github.com/roylv/avp.

## Additional files

Additional file 1: Figure S1. Phenotypic diversity between the CC lines. (*A)* phenotypic distribution among males and (*B)* females. From top-left, counter-clockwise: trabecular bone volume fraction (BV/TV; %), trabecular number (Tb.N; mm^-1^), thickness (Tb.Th; mm), and connectivity density (Conn.D; mm^-3^). (DOC 400 kb) Additional file 2: Table S1. Trait means. **Table S2.** Trait correlations. **Table S3**. Candidate genes. (XLSX 17 kb) Additional file 3: Figure S2. Haplotype mapping for the albino trait using 38 CC lines. Plots show –logP values (y-axis) according to chromosome location (x-axis). Horizontal line represent, top-to-bottom, the 95^th^, 90^th^, and 50^th^ percentile thresholds, respectively. Peak is at 7.69 –logP. (PDF 54 kb) Additional file 4: Figure S3. Confidence-interval simulations for QTL. Loci at a neighborhood of 3-5 Mb around the original locus were simulated by permuting the residual sum of squares of the related phenotype. Maximum logP was obtained along with its relative position in Mb to the original QTL (histograms, left panels), and with the number of markers from the original QTL (boxplots, right panels). Simulations results for the BV/TV loci (*Trl*1 and *Trl*2), determined with high confidence that the peak QTL is responsible for the effect seen in the haplotype scan, thus the narrow CI; *Trl*3 histogram and corresponding boxplot represent simulation results for Tb.N; plots for *Trl*4 and *Trl*5 show simulations for Tb.Th; and *Trl*6 for Conn.D. Note the narrow CI for *Trls* 1, 2 (BV/TV), 4, and 5 (Tb.Th), wide for *Trl*3 (Tb.N), and wider still for *Trl*6 (Conn.D). (PDF 113 kb)

## Abbreviations

BMD: Bone mineral density
BMP: Bone morphogenic protein
BV/TV: Bone-volume fraction
CC: Collaborative cross
CI: Confidence interval
Conn.D: Trabecular connectivity density
DXA: X-ray absorptiometry (DXA)
FDR: False discovery rate
GWAS: Genome-wide association study
H^2^: Heritability
SDP: Strain distribution pattern
SNP: Single nucleotide polymorphism
LSR: Least significant range
TAU: Tel Aviv University
Tb.N: Trabecular number
Tb.Th: Trabecular thickness
Trl: Trabecular-related loci
μCT: Micro computed tomography
QTL: Quantitative trait locus
QTN: Quantitative trait nucleotide
