# Supplementary figures and images for: Collaborative cross mice in a genetic association study reveal new candidate genes for bone microarchitecture
Source: BMC Genomics. 2015 Nov 26;16:1013. doi: 10.1186/s12864-015-2213-x (PMC4661944; doi:10.1186/s12864-015-2213-x)

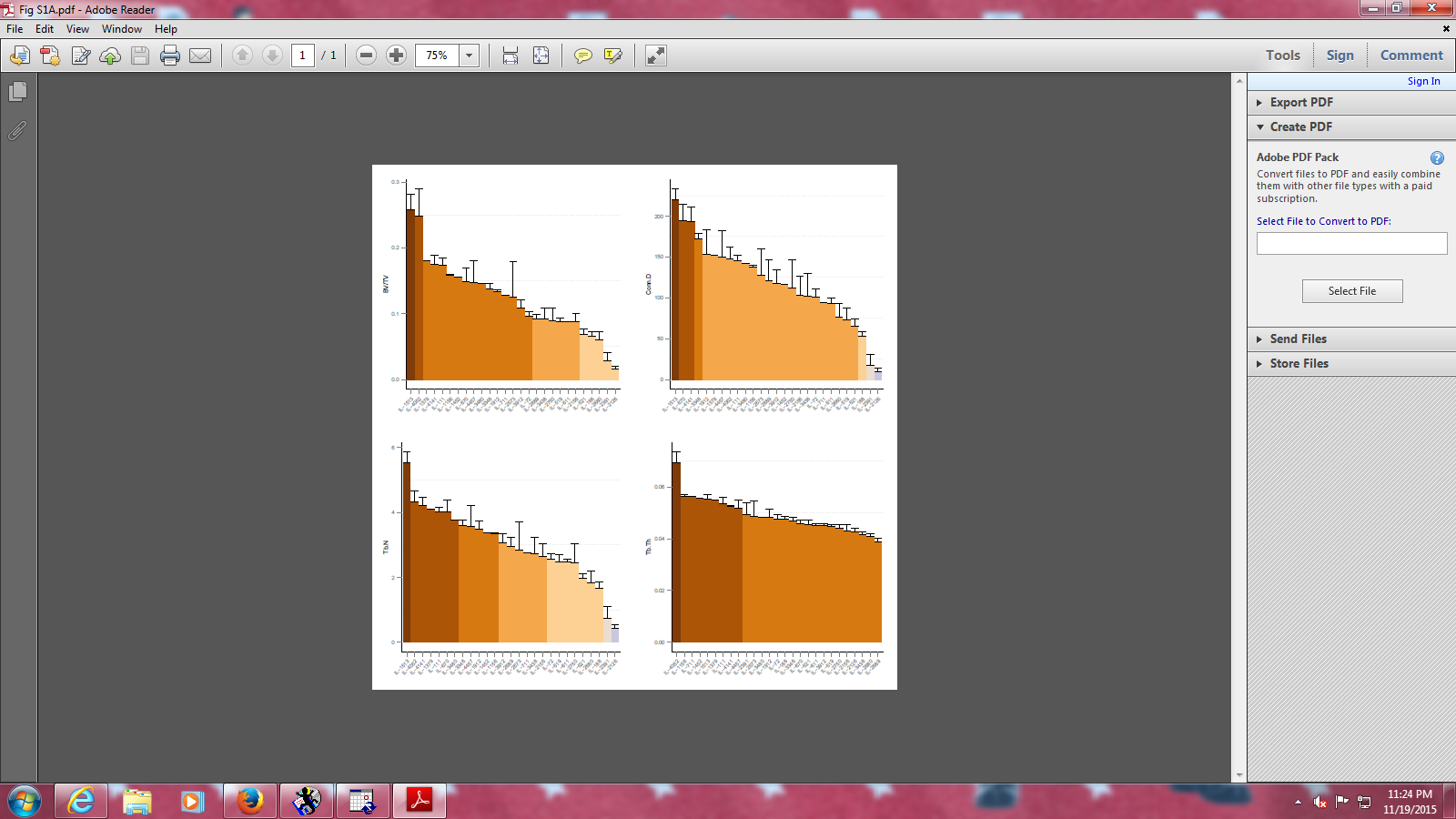


A


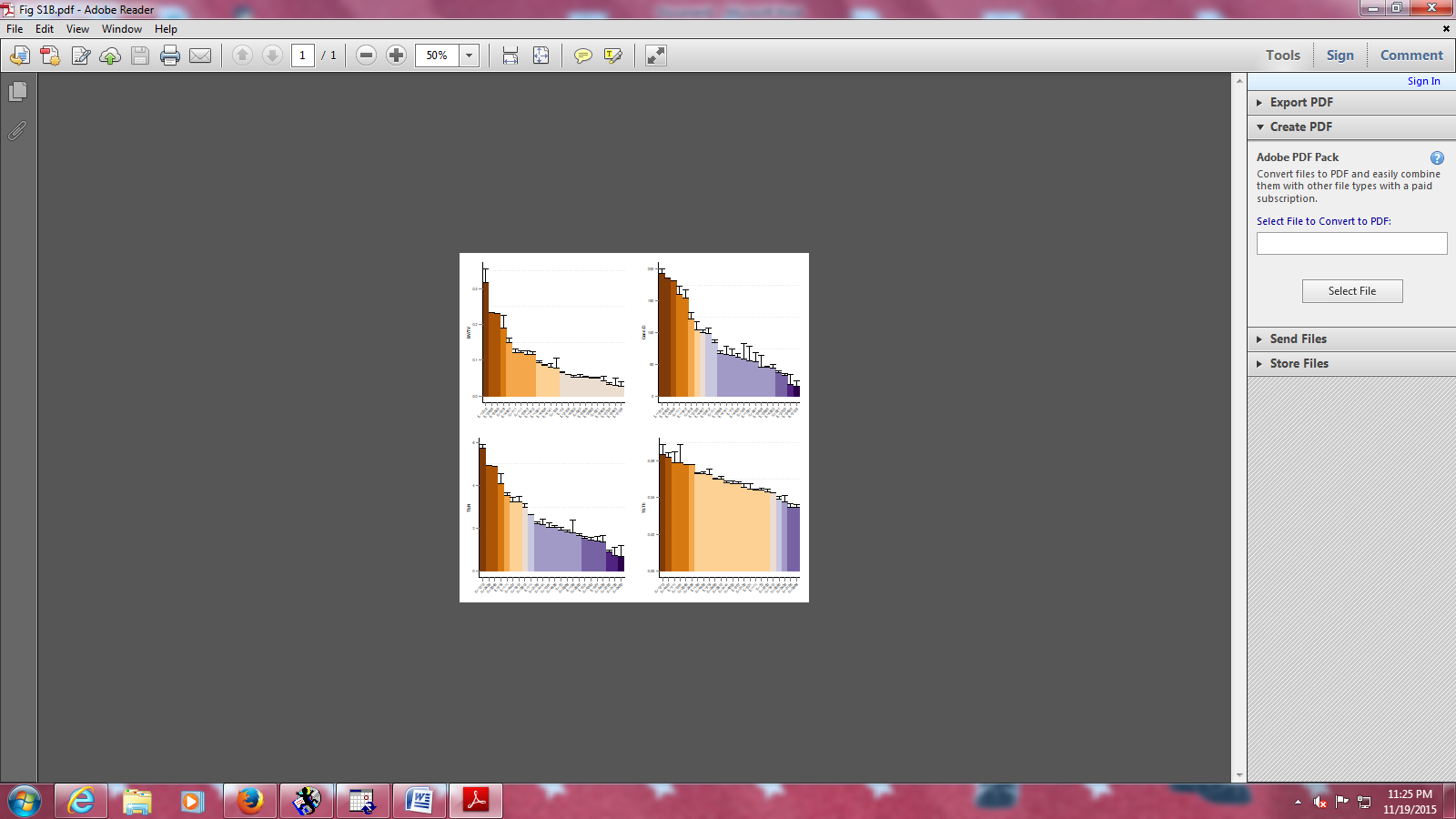


B

Supplement: Additional file 1: Figure S1. — Phenotypic diversity between the CC lines. (A) phenotypic distribution among males and (B) females. From top-left, counter-clockwise: trabecular bone volume fraction (BV/TV; %), trabecular number (Tb.N; mm-1), thickness (Tb.Th; mm), and connectivity density (Conn.D; mm-3). (DOC 400 kb) [file 12864_2015_2213_MOESM1_ESM.doc]

CcolorW

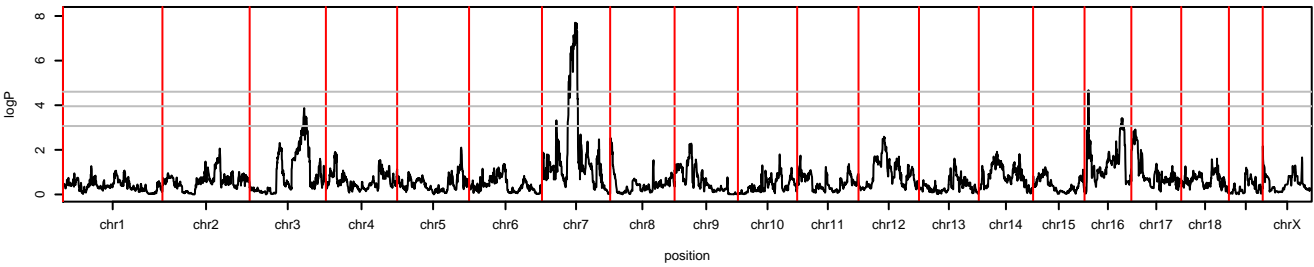

Supplement: Additional file 3: Figure S2. — Haplotype mapping for the albino trait using 38 CC lines. Plots show –logP values (y-axis) according to chromosome location (x-axis). Horizontal line represent, top-to-bottom, the 95th, 90th, and 50th percentile thresholds, respectively. Peak is at 7.69 –logP. (PDF 54 kb) [file 12864_2015_2213_MOESM3_ESM.pdf]
