# Supplementary material for: Collaborative cross mice in a genetic association study reveal new candidate genes for bone microarchitecture
Source: BMC Genomics. 2015 Nov 26;16:1013. doi: 10.1186/s12864-015-2213-x (PMC4661944; doi:10.1186/s12864-015-2213-x)
Supplement: Additional file 4: Figure S3. — Confidence-interval simulations for QTL. Loci at a neighborhood of 3-5 Mb around the original locus were simulated by permuting the residual sum of squares of the related phenotype. Maximum logP was obtained along with its relative position in Mb to the original QTL (histograms, left panels), and with the number of markers from the original QTL (boxplots, right panels). Simulations results for the BV/TV loci (Trl1 and Trl2), determined with high confidence that the peak QTL is responsible for the effect seen in the haplotype scan, thus the narrow CI; Trl3 histogram and corresponding boxplot represent simulation results for Tb.N; plots for Trl4 and Trl5 show simulations for Tb.Th; and Trl6 for Conn.D. Note the narrow CI for Trls 1, 2 (BV/TV), 4, and 5 (Tb.Th), wide for Trl3 (Tb.N), and wider still for Trl6 (Conn.D). (PDF 113 kb) [file 12864_2015_2213_MOESM4_ESM.pdf]

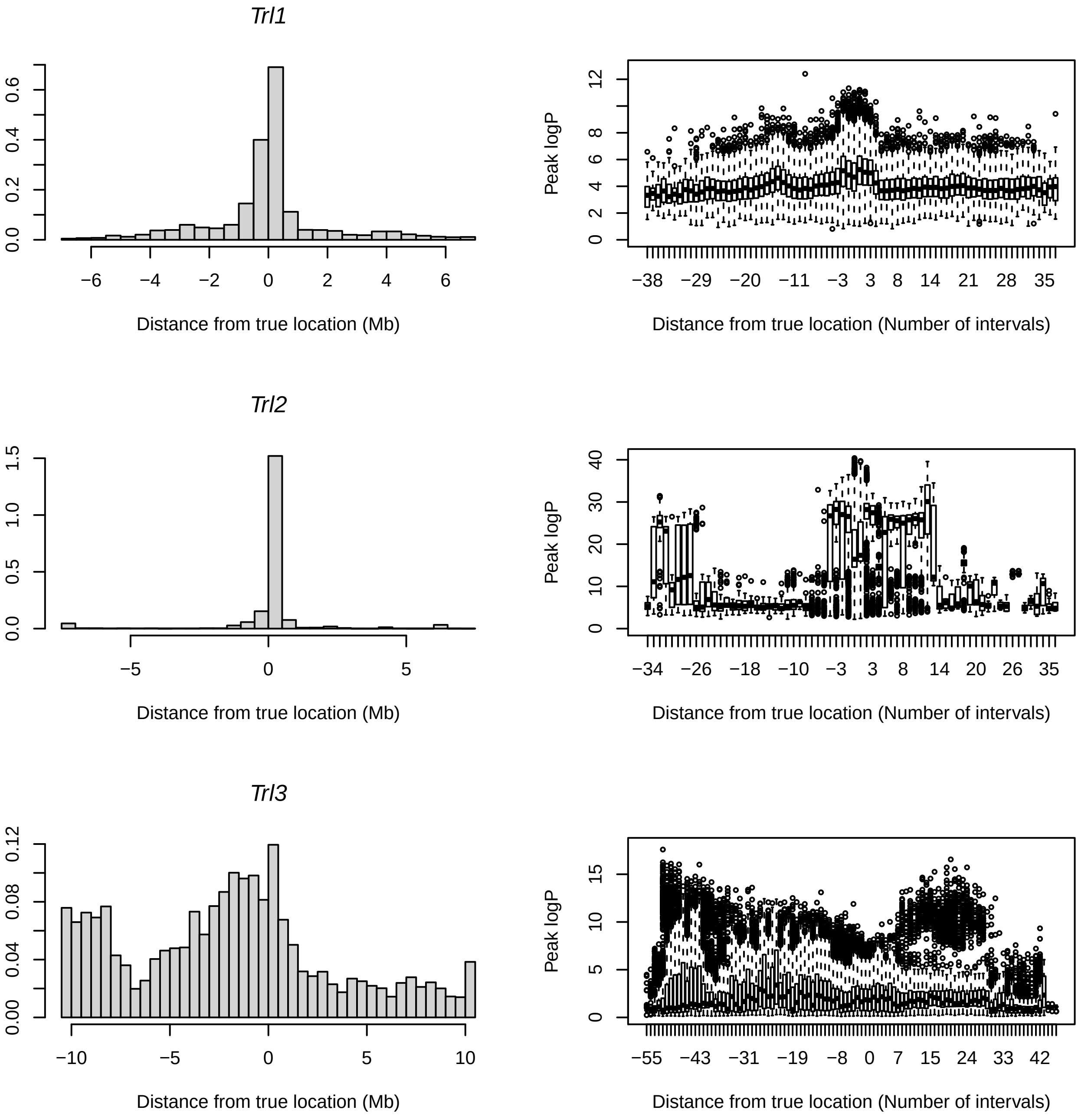
Trl1
Trl2
Trl3
Peak logP
Distance from true location (Mb)
Distance from true location (Number of intervals)
0.0
0.2
0.4
0.6
−6 −4 −2 0 2 4 6
0 2 4 6 8 12
−38 −29 −20 −11 −3 3 8 14 21 28 35
0.0 0.5 1.0 1.5
−5 0 5
0 10 20 30 40
−34 −26 −18 −10 −3 3 8 14 20 26 35
0.00 0.04 0.08 0.12
−10 −5 0 5 10
0 5 10 15
−55 −43 −31 −19 −8 0 7 15 24 33 42

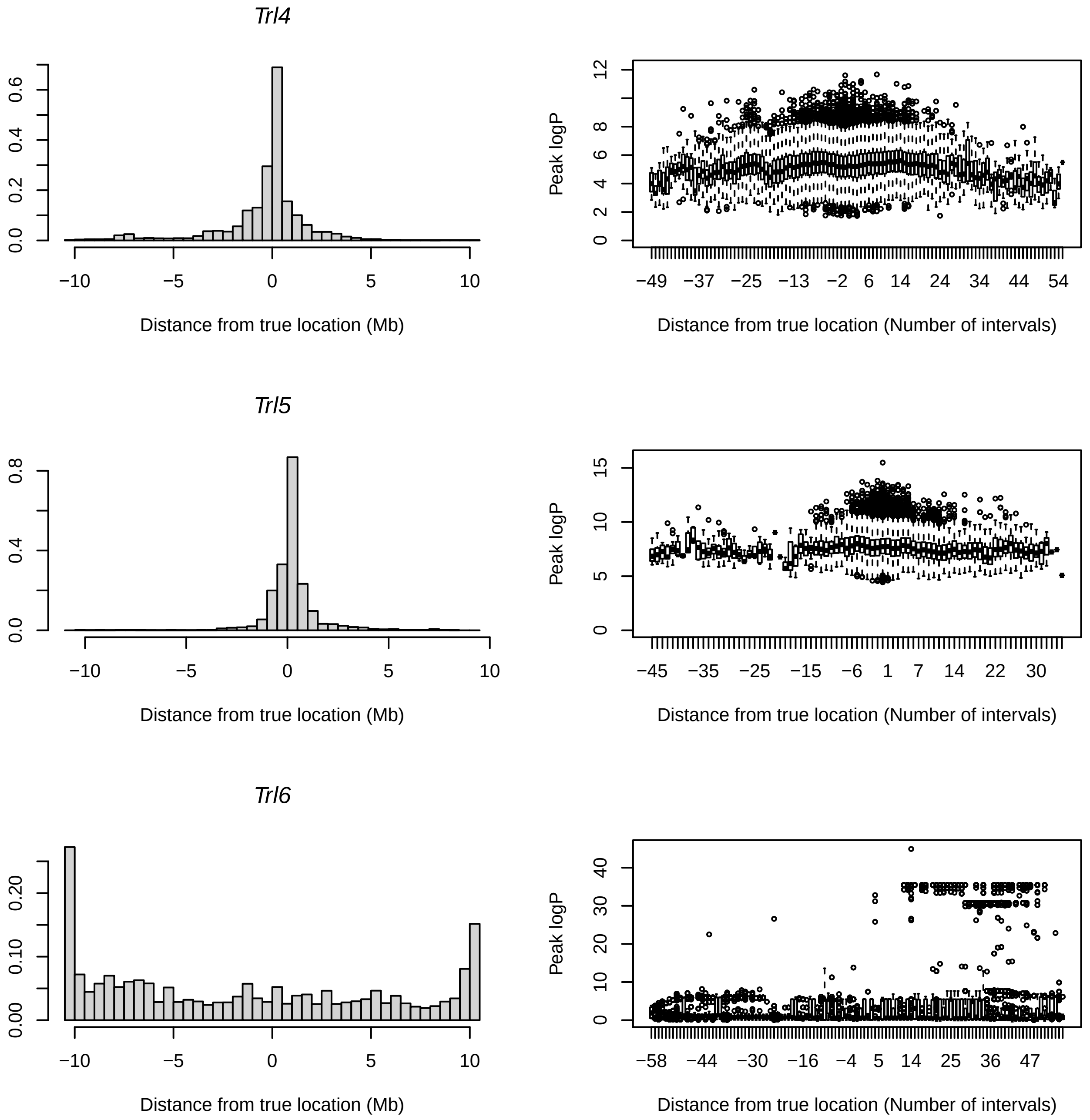

Trl4
0.0
0.2
0.4
0.6
−10
−5
0
5
10
Distance from true location (Mb)
Peak logP
0
2
4
6
8
12
−49
−37
−25
−13
−2
6
14
24
34
44
54
Distance from true location (Number of intervals)
Trl5
0.0
0.4
0.8
−10
−5
0
5
10
Distance from true location (Mb)
Peak logP
0
5
10
15
−45
−35
−25
−15
−6
1
7
14
22
30
Distance from true location (Number of intervals)
Trl6
0.00
0.10
0.20
−10
−5
0
5
10
Distance from true location (Mb)
Peak logP
0
10
20
30
40
−58
−44
−30
−16
−4
5
14
25
36
47
Distance from true location (Number of intervals)
